# Medical Topography of Brazil and Uruguay: With Incidental Remarks

**Published:** 1845-03

**Authors:** 


### REVIEWS.

---

Art. III.—*Medical Topography of Brazil and Uruguay:* with incidental remarks. By G. R. B. Horner, M.D., Surgeon U. S. Navy; Honorary Member of the Philadelphia Medical Society, and Corresponding Member of the National Institute at Washington. Philadelphia: Lindsay & Blakiston. 1845. 12mo. pp. 296.

An interesting book might be written on this subject. The countries are comparatively unexplored by medical men, and all that might be described in them as pertaining to our profession would have the advantage of novelty. The work before us, has been compiled by a young physician, from the private and public journals kept by him during two cruises, and embraces a large amount of matter having no reference to medicine. But a small portion of the work, indeed, is strictly professional, the principal part being made up of details similar to those which find a place in the diaries of all travelers. But some of his descriptions have interest for the medical man, and from these we propose to select such as appear to us to afford the most valuable information.

We begin with his description of some of the vegetable productions of Brazil, which he represents as being exceedingly exuberant. In a single acre of uninclosed land, it will often happen, he remarks, that the traveler will see more plants than he can examine carefully in many months. Of the cactuses alone, 400 species are said to be found there, growing upon the most barren rocks, as well as on the most fertile fields, in the moistest places, and in the dryest, in shade and in sunshine, but never beyond a certain elevation on the mountains. He gives the following interesting account of the banana:

"Of the banana, I will observe that three species are to be had, a small and a large yellow one; and a third larger than either, and of a reddish color. One of them is the fruit of the Musa paradisiaca, and two that of the Musa sapientium. The entire plant is termed bananeira by the Brazilians. The three species are alike, and are not easily distinguished. Each of them has a pithy, stout stalk or trunk, enveloped by the stems of its immense leaves, and the fruit of all is borne in a grand cluster, disposed in radiated bunches, inserted into the top of the stem, one above the other. As this continues to grow, flowers in half circles are displayed below its conical apex, from beneath one of the lowest leaf-like scales, half enveloping the base of the cone. Every flower becomes a banana; and after this, a scale on the other side of the cone parts from it, and simply adhering by its base, allows another semicircle of flowers to bloom; and thus a conical branch, composed of many circles or radients, is eventually formed, and bows the top of the stem so much as nearly to make it double. The banana is a most wholesome fruit to people generally; but if eaten largely, sometimes creates indigestion, and in moderate quantities produces costiveness—an effect owing to its astringency. This quality abounds in the whole plant, and having now become known to the Brazilian farmers they avail themselves of it for the cure of disease. To hasten suppuration and the pointing of abscesses, the fruit is fried, mixed with a little oil of sweet almonds, and applied as a cataplasm. The juice is most used as a remedy. It contains gallic acid, and according to two French chemists, Fourcroy and Vauquelin, nitrate and oxalate of potash, and is employed externally and internally. The complaints treated with it are leucorrhœa, prolapsus ani, atonic wounds, aphthæ, swelling of the legs and testes. For the cure of leucorrhœa it is employed thus: A stalk is divided at the middle by a horizontal cut; the upper end of the part remaining in the ground is scooped out and filled with sugar; this becomes saturated with the juice, in a few hours forms a syrup, is taken out in any suitable vessel, and given in a dose of a tablespoonful three times a day, or oftener according to circumstances. In treating prolapsus ani and hemorrhage from the rectum, it is used in the form of a clyster or lotion in town, but in the country the people, when affected with the former complaint, cut off the bananeira in the middle, sit upon the stump, and allow the affected part to remain from a quarter to half an hour in contact with its upper surface."

He gives a brief but interesting account of the coffee-tree which we extract:

"Of coffee I also will speak briefly, as its history is known generally. Most of it and that of the best quality grows upon high grounds, the sides of hills and mountans, up to a certain height, but does not prosper on the summits of even those of medium height, from the coldness of the air upon them. I have not seen it growing more than two or three thousand feet above the level of the sea. After the ground has been prepared, by cutting down trees and bushes and burning them, the coffee scions are planted in rows. These are near together; only enough space is left between them and the scions to allow the trees to attain a full size, and the fruit to get ripe. When so, it is blood-red, rather less than an inch long usually, and of half the diameter. It has a thin, pulpy skin enclosing the kernel, has a sweet, gently astringent, and aromatic taste, and is a favorite food for children. Many coffee trees grow along fences, and attain there the largest size, but the highest do not exceed fifteen or twenty feet. When the fruit is mature, and often when not, it is plucked, spread out to dry on rocks and scaffolds, then pulled and sifted: now and then it is allowed to get wet with rain, looses its greenish color, is not so plump, and less valuable in market; but by many of the best judges of it, is more highly esteemed than the green. Of the two kinds more than a million of bags are exported annually from that country, and its inhabitants consume many thousand bags besides. The total amount of coffee produced in Brazil in 1843, is estimated to have been the enormous weight of 170,000,000 of pounds, or about a third of what is produced in the whole world."

But we leave these subordinate matters to extract the account given by the author of the *Organization and Statutes of the Medical Schools* of Brazil, which, we are sure, will be new to most of our readers. These are two in number, one of which is situated at Bahia, and the other at Rio. The latter is the principal one, and Dr. Horner gives the following description of it:

"Shortly after King John VI. fled from Portugal, and in the year 1808, he established in Rio a school of anatomy, surgery, and medicine. This school continued until the organization of the college of medicine and surgery by Pedro I, September 20th, 1826; and in October, 1832, the year after his abdication, the latter school was abolished in name, and reorganized under its present title: Escola de Medecina de Rio de Janeiro. This change was effected by the regency of the Empire during the minority of Pedro II.

The faculty of the school are the following physicians, viz:—

*Director.*

Manoel de Valladao Pimentel.

*Lent Proprietors, or Professors.*

First Year.

F. F. Allemao—Botanical Medicine and Elementary Principles of Zoology.

F. de J. Candido—Physical Medicine.

Second Year.

J. V. Torres Homem—Chemical Medicine and Elementary Principles of Mineralogy.

J. M. Nunes Garcia, (*Examiner*)—General and Descriptive Anatomy.

Third Year.

D. R. dos G. Peixoto—Physiology.

J. M. Nunes Garcia—General and Descriptive Anatomy.

Fourth Year.

J. J. de Carvalho—Pharmacy, Materia Medica, especially Brazilian, Therapeutics, and the Art of making Formulæ.

J. J. da Silva—Internal Pathology.

L. F. Ferreira—External Pathology.

Fifth Year.

C. B. Monteiro, (*Examiner*)—Topographical Anatomy and Preparations.

F. J. Xavier—Midwifery, Diseases of Pregnant and Lying-in Women, and of Infants recently Born.

Sixth Year.

J. M. da C. Jubim—Legal Medicine.
T. G. dos Santos—Hygiene and History of Medicine.
M. de V. Pimentel—Internal Clinics and relative Pathological Anatomy.
M. F. P. de Carvalho—External Clinics and relative Pathological Anatomy.

*Lent Substitutes, or Assistant Professors.*

| | |
|---|---|
| A. T. d'Aquino,<br>A. F. Martins, | Section of Accessary Sciences. |
| J. B. da Rosa, (*Examiner*)<br>L. de A. P. da Cunho, | Medical Section. |
| D. M. de A. Americano,<br>L. da C. Feijo, (*Examiner*) | Surgical Section. |

*Secretary.*

Dr. Luiz Carlos da Fonseca.

"Besides the above persons employed in the school, are a librarian, treasurer, porters, messengers, and servants.

The course of instruction begins on the 1st day of March and ends on the 20th of December; on which days the whole faculty meet. They likewise do so whenever the director thinks it necessary, and summons them. He is elected by the faculty from among the professors. Some of the substitutes are allowed to vote for him, but none of them can be a candidate. The ex, or pensioned professors, when invited by a majority of the faculty, can deliberate with them, and are entitled to attend the meetings at all times. The meetings are ordinarily secret, and continue two hours; and the acts of the faculty, when exigency requires, are also kept secret. The faculty are empowered to judge of violations of the statutes of the school by any of the members, students, and persons employed in it; and before consideration of the case is made, may allow eight days to collect testimony. Should the director be guilty of misconduct, the faculty, when it is recommended by a committee of three professors, and the proposal has been taken into consideration, nominate another committee of three, to examine into the matter; and if the director be found guilty, his misconduct is made known to the government. Should he be proved not guilty, he forthwith resumes the discharge of the duties of his office. Should he not be acquitted, and the case be referred to the government, it may reinstate, suspend or dismiss him.

"Every professor receives a salary from government of 2,000 milreas* per annum; the substitutes receive 1,200, and the director 3,000 milreas, as he acts both in the capacity of president and professor. and has the supervision and administration of the school. After twenty years service every person can retire on a pension for life; and before the expiration of that time, should bad health prevent a further discharge of the duties of his office.

"Professors and substitutes after twenty years, counting from the date of their nomination, receive pensions for life equal to the whole amount of their salaries. Should any one of them, from age or sickness, be incapaciated from duty within the first ten years of service, he receives a pension amounting to a half of his salary; and should he be disabled between ten and twenty years of service, his pension is protionately increased. Another very liberal provision is made for the faculty. If any one having completed his twenty year's service desire to continue on duty, he can do it with the authority of government, and is given an increase of a fourth of his salary; and if he be able at the expiration of four years to discharge his duties, he continues to do so upon being again authorized by government, and during the second four years receives another fourth part. The same is done at the expiration of eight years, as long as the professor or substitute continues in office; and when at last he is no longer able to keep in it, he goes into honorable retirement, and has a pension equal to the whole amount of salary received at the time of resignation.

"Rank among the faculty is determined by the dates of nomination, or by the time they entered into office, and when they were nominated at the same period, the person takes rank who first received the degree of doctor. None others than Brazilians can be appointed members of the faculty.

"Exchange of chairs can be made by them with the approbation of government, after the faculty have taken the exchanges into consideration and determined that they would be beneficial to instruction. When it has been determined that an exchange may take place, the decision is sent to gov-

* About $1,100; the value of the milrea being reduced to about 55 cents. At par it is worth about 100.

ernment to receive its confirmation; and when it is made the exchange is published in a newspaper. In case of a chair being vacated by death, retirement, suspension, or dismissal, the same form as the preceding is observed; the person thought competent is nominated by the faculty, and if approved by it, receives his title from government. To fill vacancies the school is granted three honorary professors and six honorary substitutes. In appoinments to the former distinction, substitutes who have served twenty years have the preference; and for the honorary substitutes doctors of medicine who desire to travel at public expense for scientific information, and gives satisfactory proofs of merit, and scholars of the practical school* who have received six premiums during the session, are preferred. The honorary professors and substitutes as well as the effective ones receive their titles from government. Substitutes of the former kind can confer with the latter, and supply their deficiencies in teaching without taking an active part in council.

"For an effective substitute three kinds of election are held: one for the accessary sciences; a second for surgery; a third for medicine. A candidate for the place must be a citizen of Brazil, and in the enjoyment of civil rights. He must also show a diploma conferred or approved by one of the faculties of Brazil, and produce testimony of good habits, attested by a justice of the peace in his district. Within eight days after the vacancy occurs, the director having received the approval of government, makes known by advertisement that the chair is vacated, and an election to fill it is to be held, and mention the qualifications required. Six months are allowed to furnish them; and within that period applicants have to forward the necessary documents; and such as are permitted to be candidates are enroled by the secretary, and have their persons identified by the director. Fifteen days before the expiration of the six months the secretary announces that the enrolment will be ended on the first day after the above period is terminated; and when this has hap-

* The practical school is nothing more than a part of the school of medicine, but is formed of another faculty; composed of a regent and 12 pensioners, with any number of scholars, who assist the professors in making preparations, dissecting, and experimenting in chemistry, pharmacy, &c., &c. The regent has a salary of 800 milreas for his services; and the pensioners 200 each. By pensioners here we are to understand simply graduates of the school of medicine, employed and paid by government, and not ex-professors drawing pensions.

pened he sends a list of candidates to every member of the faculty. Three days subsequently to the closure of the list a council in secret session is held, the list is read, and an examination made respecting the morality of the candidates. A secret vote is then given on them for their admission or rejection; which last is the lot of him who does not receive two-thirds of the votes of the members present. The director after the session gives an account to government of the decision of the faculty. Should no candidate be admitted, another announcement of the vacancy is made, and a period of three months more allowed the applicants to furnish the necessary credentials.

"The faculty are the judges of the election; but no member can vote who is the father of a candidate, or his relation within the second degree of consanguinity. The director acts as president of the jury, as it is called, watches over the mode of election, gives admonitions if order be not observed and can suspend the proceedings and cause the expulsion of the offender should he be a candidate, an alumnus of the school, or a stranger. The session may be continued or deferred after a new deliberation, and the director reports the whole affair to government. After preliminary proofs of merit have been given, the election of candidates proceeds; and they have to furnish actual evidences of their professional qualifications and ability to discharge the duties of the vacated chair. The first evidence is a written discourse in the presence of the jury on one of four subjects, selected the evening before the election by a committee of three members, and placed in an urn, after being read to the board. The subject drawn is the one on which the lecture must be written; and this must be done on the same day. The candidate also has, within twenty-four hours after the lot is drawn, to deliver an oral discourse upon the subject named; he must, moreover, deliver a thesis on some professional subject, and give practical proofs of being qualified for the place he seeks, and especially with regard to the subject of the thesis. On a fixed day the candidates in succession ascend the magisterial chair of the hall and deliver their written discourses, and three days after them the oral ones. At the expiration of three months from the last of the latter, the examinations upon the theses are made, and the candidates are held responsible for the moral and legal sentiments expressed in them; and forty-eight hours before the recitations they have to furnish sixty copies of their theses for the use of the faculty and for other purposes. After the candidates have read and defended their theses, the professors examine them upon them until satisfied, and then proceed to ballot; first, to decide whether the candidates be worthy of the appointment; secondly, which of them is most worthy. If there be one candidate only for the place of a substitute, a majority of one elects him; if there be two candidates the one who has the plurality of votes in three ballotings, becomes the substitute. When the election is decided, no objection on the part of the candidates is noticed, unless some informality occurred, and in this case the matter is reported to government. Should it ordain a new election, it is held, and the same candidates are taken into consideration by the faculty. But no election for the professor is held, if there be only one effective substitute who seeks it, nor does the election take place if there be not present in all the sessions, at the time the election should commence, the legal number of effective substitutes. Other modifications are made in the election of a professor;—first, effective substitutes are permitted to become candidates, and when the former are wanted, honorary substitutes are taken. Secondly, the period for qualification is only two months, the morality of the candidates is not previously judged, the written discourse is upon twelve questions, six of which relate to the different branches of medical instructions; and if the election be for a vacancy in a clinical chair, the oral discourse will be made on patients, designated in the hospital of Miseracordia.

"Finally, with respect to elections, I will state, that if they be for a physician to travel at the expense of the State, they are done in conformity to law, and after the manner determined by the faculty; but not before notice has been publicly given for twelve months previous to them, and the conditions of the travel have been made known at the same time.

"But the examinations are not confined to the professors, substitutes, nor their pupils. Every graduate of former Brazilian schools, before he can receive a title or diploma, has to present to the director such diploma, must identify his person, show the receipt of the treasurer for the matriculation fee, and the certificate of the same that the applicant has deposited with him the practical observations required. Native and foreign practitioners are obliged to produce certificates of identity and naturalization; and practitioners settled in the country previous to a resolution adopted October 27th, 1835, are required, if they wish to continue in practice, to produce the above documents, and likewise their passports, or the certificates of their consuls, signed by the police of their place of residence in Brazil, declaring the day, month, and year of their arrival in the country.

"All physicians, surgeons, apothecaries and midwives who have settled in it the above time, or who may hereafter do so, must, before they begin practice, undergo a theoretical and practical examination. These examinations are conducted as those of the school—by two professors and a substitute, save that respecting the thesis; this examination will be made by three professors and two substitutes; but the faculty are empowered to dispense with the thesis, and establish any other proof of competency. Surgeons are first examined on anatomy, external pathology, and parturition; and secondly, are clinically questioned on two surgical cases, and obliged to perform any operation required. An equally strict examination of physicians takes place; and apothecaries are required to prove their knowldge of the materia medica, and then make the pharmaceutical preparations determined upon by the faculty. Should the examinates be approved, the director confers the same titles as on the graduates of the Fluminensian school; but if the examinates are rejected, they lose the fees deposited, and have to undergo another examination, after the same fees have been again paid, and six months have elapsed.

"From the above facts, it is evident that Brazil, though far behind the United States and other countries in most respects, has some excellent laws relative to its faculty. She affords the greatest encouragement to native talent and professional attainments, and does not allow herself to be inundated by crowds of empirics and illy educated members of the profession of medicine, who may be attracted to her shores by hope of gain, or of a reputation to be attained by their imposition, and then maintained by the credulity of the people. In this restraint on adventurers, the Brazilians consult not only the true interest and happiness of themselves at large, but especially benefit the faculty of the empire; and, moreover, protect from extortion and unnecessary pain such of its subjects as are poorest, and most apt of all others to be duped by either foreign or domestic empiricism. No cry in Brazil is heard against monopoly in the practice of the healing art, by its confinement to a few persons; nor do the faculty take advantage of such monopoly, and charge exorbitantly for services. On the contrary, they leave them to be valued by the patients, do not send in bills,* nor employ collectors. Of course, whatever reward they receive proceeds from a sense of duty and obligation, or from gratitude on the part of the sick or their friends. For exorbitant fees then they are solely to blame; and it is not probable that these occur often unless the Brazilians are different from most nations, and voluntarily reward physicians more liberality. Their custom of leaving their fees to the means and generosity of patients, no doubt influences them in paying larger fees than they would willingly if bills were presented; but, nevertheless, the incomes of physicians, including every branch of the healing art in Brazil, are not generally large; none that I have heard of are really great and the profession of medicine in the empire is neither distinguished for wealth, nor display in dress, equipages, fine dwellings in the city, nor gorgeous residences in the country; and though highly respectable, are not as fashionable in Rio Janeiro as in some of our Atlantic cities; nor, indeed, are they likely to prosper much should as great odium be excited by them as recently by a member of the profession, whose name remains unknown to me, but has the credit of killing a patient in September, 1842. Great was the excitement caused by the reports regarding the case. The family of the deceased were kind and generous enough to conceal the name of the physician, but the prescriptions were obtained in some manner and published. The last and finishing doses were—8 grs. of sub-nitrate of bismuth, given, with several less active articles, on the 13th and 16th; and these not having the desired effect, the following were presribed to be given from 2 o'clock, P.M., to 12 o'clock at night:

℞. Infusion of lime tree, ℔ii.

Tart. emet., grs.ii. M.

Next came tart. emetic, grains *twenty*, divided into 4 parts, and lime tree electuary, 3 ounces, taken with oil of croton tiglium, 5 drops. The parturient potion of Velpeau was afwards given, and then the patient was drenched with orange, distilled, and barley water, and took an ounce of the syrup

---

* This statement is inapplicable to the English physicians at Rio. They follow the customs of their country, have specific fees, and send in bills generally. The common fees for a visit is two milreas in the city, and four in the country, and ten milreas for attending a consultation. Among people well off, the price of an obstetrical case varies from 50 to 100 milreas, or from 30 to 60 dollars according to their present rate.

of orange-peel. It is hardly necessary to state, that the first named prescriptions were very active if not efficacious, and the patient was vomited and purged into the next world by daylight on the morning of the 17th.

"To continue my remarks on the faculty of the school, I will observe that they exercise entire control over the students within the precincts. Should one of them during a lecture or at another time disturb the peace, the professor can command silence; if he should not obey, the order is repeated at the same moment that the offender is called by name. and pointed at. Should this be insufficient, he is commanded to retire, or the professor leaves the hall and reports him to the director. Besides penalties incurred immediately for misconduct, the students are liable to undergo others at the end of the session. At this time any professor and substitute is required to present to the faculty a tablet of observations made on the conduct of students, and to confirm them by facts. The secretary keeps those tablets in the archives, where they are not allowed to be examined without the consent of the director, and are retained for the use of the faculty and government when occasion may require. The faculty themselves are liable to punishment for misdemeanors, such as offences against public morals, neglect of public duty, and conduct notoriously scandalous and compromising their honor. A professor, for instance, who, three successive times, and without the consent of the director, or without just cause, should fail to discharge his duties by not lecturing, is liable to be brought before the academical council by the director or three professors, and to be fined in a sum equal to his salary during the days he has not performed his duties.

"The statutes with respect to students are precise. Offences are strictly defined, and severely punished when aggravated; and for the information of the students the penalties to be incurred are written down opposite acts pronounced offences. Thus, non-attendance at a whole lecture incurs one point or mark from the professor or substitute in the chair; making a noise, especially during a lecture, incurs one or two points; non-attendance to an examination is noted down, and punished by the offender being examined last, and fined ten milreas; departure before the end of a lecture and scholastic exercises requires an explanation, and receives one point from the professor present; irreverence, tumult, lampoons, caricatures, obsceneness, acts of cruelty towards the patients, offences against public morality or modesty, libels, disobedience to the order of the director, mockery and ridicule of the professors, substitutes, or attendants of the school; any attack whatever on them, any marked insubordination, affronts to fellow-students in the precincts of the school or without them, are punished by points and reprimands, and by temporary or permanent expulsion from the school, after the government has been informed of the circumstances and has given its approval."

He adds to this an account of the ceremony of conferring the diplomas upon the graduates, the conclusion of which is striking. The address of the president, and of a graduate who follows him, over, and the medals, bouquets, and diplomas, being distributed, an oath follows:

"The oath taken was the following: 'In the presence of the masters of the school and of my fellow disciples, I—F. H. de Mouro—swear on the Holy Evangelists, that in the practice of medicine I will be faithful to the laws of honor and probity.' Then he places his hand on the works of Hippocrates, and continues: 'I promise on the works of Hippocrates that practising into the privacy of families my eyes will be blind, my tongue will keep the secrets confided to me; never shall my profession serve to corrupt good manners nor to favor crime. Grateful to the masters who instructed me, I will honor them in life and respect them in memory. I will guard, as my own, the honor of my colleagues, and will never deny them the fruit of my experience. The indigent shall never cry in vain for the succors of my art. I will employ all my zeal in the diminution of their sufferings. Let men recompense me as they think proper if I should be faithful to my promises; and let me be covered with opprobrium and disrespect by my colleagues if I should ever be wanting to them.'

"The oath taken, the president continues the ceremony by saying, 'Read and meditate on the works of the father of medicine. Regulate your life by him, and men will cover your name with blessings.' The president then hands the graduate a volume of the works of Hippocrates, thrusts a ring on his finger, and says, 'Receive it as a symbol of the degree I confer; you can practice and instruct in medicine.' Having pronounced these words, the president embraces the new doctors, and, as no one succeeds him, here ends the ceremony. When this happened bouquets again were distributed, the assembly displaying their trophies dispersed, and the canon

Januario, the venerable hoary-headed president of the National Library, known by a dazzling star of diamonds on his left breast, made off with one of the largest and handsomest bouquets. He looked as pleased as a bridegroom just blessed with a wife, and, though a bachelor, reminded me vividly of the marriage of Pope's January and May, when I saw his grey head and fine flowers and was told his name."

A notice of the hospitals at Rio follows his account of the medical school. Here he saw numerous cases of leprosy, or elephantiasis, as it is differently called, of which he gives a graphic description:

"Of the nature of this disease I will speak another time; and now will merely state that I found twenty-five females and seventy-three males in the hospital affected with it, and most of them were adults; among them were some most distressing objects. Frequently one part of the body had become preternaturally enlarged while another had wasted away. Some had lost their noses, while the lips had grown to a great size, or the ears had attained almost that of a young elephant's. Others had the whole face enlarged, their legs swollen and covered with scales; while another set had lost an eye, and had it covered by a fleshy substance like fungus. A black woman had lost one foot and all the fingers, except the first phalanges, and these were flexed on the palms as if burnt by fire. Such was my impression at first sight, but on close examination and inquiry I detected my error. Several had no nails to their fingers, and these were gradually decreasing, though no sign of ulceration was perceptible. In fine, notwithstanding a number of miserable beings had their limbs enlarged, none of them had their bodies really corpulent. On the contrary, they were commonly emaciated, and indicated the want of sustenance, rather than a superfluity."

In another place he continues:

"Elephantiasis exists in every form and degree; spares no sex, age, nor condition; pays no respect to natives or foreigners; affects the poor and rich; harasses servant and master; affects the plebeian and patrician. The poor and laboring classes, however, are most annoyed by this disease; and the negro population, both the slaves and free portion, have a full share. The persons most affected with elephantiasis, strictly speaking, and according to the derivation of the name, are those who live miserably, or are obliged to make much use of their feet, and have them frequently, and for a long time, exposed to the sun without shoes or anything else to protect them. One of the best evidences of this fact was, that I knew a sailor to bring on a violent inflammation and swelling of his feet similar to elephantiasis from such exposure. Had the irritation been kept up, I have little doubt a chronic affection would have been induced, and a permanent enlargement taken place. I have observed, also, that the laboring class of people, particularly men who stand or walk much, and women who are in the habit of washing clothes and immersing their hands in hot water or other warm fluids, have them or their forearms affected."

"Of the two forms of elephantiasis it is not easy to say which occasions the most inconvenience to the sufferers; for one lops off his members, the other enlarges them to an enormous extent; and both deprive of the means of obtaining a livelihood; one by a deficiency, the other by an excess. A seamstress and shoemaker lose their fingers by one form of the disorder; a footman and porter have their legs and scrotum so much increased by the other that the former can only stand, and the latter has as much as he can carry about his own person. A belle, ambitious of having the smallest of feet, the most delicate of ankles, has them converted into stumps, or such heavy clubs, that all elasticity of gait is destroyed; and so far from dancing well, cannot walk with common grace. A beau, desirous of displaying his finely-formed features, and causing every belle to look with admiration at his noble Roman nose, his expressively dark eyes, has the former converted into a genuine pug, or insignificant Grecian; and only one of the latter is left to view the ruins of the once sublime bridge, and the extinction of its fiery fellow orbit.

"Cases of scrotal elephantiasis are nearly as numerous, if not quite as much so as those of the feet; and the first attain the greatest magnitude of any other form. One or more instances have occurred where it was so large that a wheelbarrow was requisite to carry it; and two cases I saw of it were about four feet in circumference. One of them was that of a negro in the Miseracordia; another that of a late officer of customs, who resides in the Rua dos Bazotes, or Busson's street, which takes its name from the number of such persons residing on it. Dom Francisco H. C— had been afflicted with the complaint only three years, and he, nevertheless, had the scrotum formed into a vast tumor of nearly the natural shape of the part. The skin was quite firm, smooth, of a reddish-white color, and felt soft, but was firm and elastic; the prepuce spread out six or eight inches around, and divided into irregular lobes, semitransparent, fungous, and entirely concealing the glans. This was embedded in the mass. His feet and legs just above the ankles were correspondingly enlarged. The first were too big for shoes, and protected by moccasins; the second were bottle-shaped, and a linen sac supported by straps held the unwieldy scrotum. Dom Francisco is no longer able to obtain a livelihood by labor, and receives alms privately. His age is fifty-six years; and as his chin and face are becoming affected more and more, he must expect to spend the rest of his life in misery beneath the weight of his irremediable disease.

"The treatment adopted to cure this or other forms of it, is illy defined and unsuccessful—a mishap to be expected, since the causes are continued, and the local complaint often depends on a constitutional affection. Lotions are the chief medicines employed, and excision has been occasionally practised. A scrotum, weighing, it is stated in the Fluminensian Medical Journal, 143 pounds, was removed, and the patient, at the time of the publication, was in a good condition. Whether he got well, is not mentioned by the journal, but I have understood he died from the operation. This has been since repeatedly performed, and it is said with success in some cases. Hence the operation can be properly recommended when medicines fail, and the disease progresses, provided the tumor has not attained as great size as above-mentioned; for its removal then requires a more extensive wound than the patient would probably be able to survive; and even if the tumor could be excised with perfect safety and certain success, it is extremely uncertain that the Bazotes would subject themselves to a long and very painful operation, and, moreover, pay a high price for removing a natural *card-table*, besides being put to the expense of buying an artificial one afterwards. This would be right-down extravagance; as it is stated, on good authority, that some of those affected with this form of elephantiasis are in the habit of sitting with the tumors before them, and using them instead of tables in playing cards. For this purpose a stool is placed under each tumor, unless rendered unnecessary by the owner sitting on the floor, or on a counter. The shopkeepers, least of all other persons, would allow the operation, when it would occasion the loss of an article of furniture so fashionable among them, and especially as it causes them little inconvenience, is seldom in their way from their sedentary habits, and can be so easily kept upon or behind their counters, accordingly as may be necessary from the presence of male or female customers."

The climate of Brazil is a remarkably equable one. During 208 days, according to a register kept by the author, the range of the thermometer was only 25°. The period extended from June to January, in which time 63°, Fah., was the minimum, and 88° the maximum. This was on board the ship; on shore the temperature was several degrees higher. But of the climate of Brazil, says Dr. Horner, taken altogether, "it may be safely asserted that it is not excelled by the climate of any other country in the world of the same extent; and though it may be too warm for health or comfort at certain seasons, yet it is mostly unsurpassed in salubrity and agreeableness."

Our author describes Uruguay as very salubrious. He says:

"It is singularly exempt from fevers and other complaints caused by malaria; bilious, remittent, and intermittent fevers are scarcely known, and in the capital are said not to exist. Exemption from these diseases may be properly attributed to the want of marshes, swamps, and stagnant water, the undulating face of most of the state, and the dryness, if not the singular composition of the soil, and the nature of its productions. Rank vegetation is seen no where, and plants as well as dead animals parch up but do not putrify.

"A form of typhus fever, termed febre cerebral by the citizens, prevails sometimes at Montevideo, and in 1838 destroyed many of them treated after the ordinary method pursued there. Venesection proved a fatal remedy, and Dr. Carduret, convinced of this fact, forbid it altogether, resorted to less debilitating means, and acquired great celebrity by his uncommon success. I was told by a member of a family in which he was employed that he excited thereby great jealousy among his professional brethren, and from misrepresentations about him he was put in prison, and when let out forbid to practice until by the efficacy of his prescriptions to patients who flocked to his house, as he could not go to their residences, he convinced the public of his merits. He was then permitted to practice again wherever called, and now enjoys both a high reputation and a full purse."

Physicians he represents as being scarce—

"The greater part of Uruguay is wretchedly supplied with practitioners in medicine, and it is still worse off for those in surgery. The most illiterate quacks are the sole members of the healing art in many places, and even in the national army a want of well educated medical men exists to a great extent. According to what I could learn, it has no regular medical corps, and goes into battle destitute of even the most incompetent surgeons. A gentleman informed me he saw the stump of an arm, shot off in a battle, dressed and healed with cow-dung; and an old Gaucho present, when he told me this, stated he knew a soldier who received five lance wounds in the body, and had the whole of them cured by the application of the above sovereign remedy, kept moist by the urine of the same valuable domestic animal from which it is procured, being poured through the cloths enveloping the poultice and patient."

As the fees are high, here would seem to be an opening for some of our young graduates. Dr. Peixoto's charges for operations, according to his published schedule, are as follows:

"For trepanning he charges $100; for tying the carotid artery $500; for lithotomy $1,000; for the Cæsarean section 2,000 patacons."

The climate of Uruguay is subject to greater variations than that of Brazil. The range observed by our author was from 42°, which was in August, to 81°, which occurred in January. So dry is the atmosphere, he observes, "that twenty carcasses do not create more disgust than a single one in the United States." He adds:

"Any dead animal thrown into a place where the sun or winds can reach it, in a very short time becomes deprived of its juices, and has its skin and flesh made as dry as parchment. During the prevalence of the north and northwesterly winds the greatest drought occurs, and they are said to have blown much in 1831, '32, and '33, when no rain fell at Buenos Ayres; the pampas back of that city were stripped of vegetation, the soil was converted into dust, the sun totally obscured by clouds of it floating over the city, and deceiving the people with expectations of rain."

"The north wind at Buenos Ayres is well known to cause a peculiar headache, attended with excitement of the brain, and a disposition to rage and combat. An observance of this fact, I understand from high authority, caused Governor Rosas never to engage in fight with the Indians of the pampas while this wind blew, for he had found them always more ungovernable then, than when it was southerly."

Some other extracts we should like to make, but find that we have already taken up as much room as can be given to this work. Upon the whole, we have no hesitation in saying that we have found it entertaining. The author attempts nothing in the way of fine writing, but tells his incidents, and makes his comments in simple language. If this is sometimes incorrect, it is at all events unambitious, and it may be added, that the diary of a surgeon on board a man-of-war is not the place where we should look for finished composition. Y.

